# The microbiome of diabetic foot osteomyelitis

**DOI:** 10.1007/s10096-015-2544-1

**Published:** 2015-12-15

**Authors:** S. A. V. van Asten, J. La Fontaine, E. J. G. Peters, K. Bhavan, P. J. Kim, L. A. Lavery

**Affiliations:** Department of Plastic Surgery, University of Texas Southwestern Medical Center, 5323 Harry Hines Blvd., Dallas, TX USA; Department of Internal Medicine, VU University Medical Center, Amsterdam, The Netherlands; Department of Infectious Diseases, University of Texas Southwestern Medical Center, Dallas, TX USA; Department of Plastic Surgery, Georgetown University Medical Center, Washington, DC USA

## Abstract

The purpose of this investigation was to evaluate the diversity of bacteria in diabetic foot osteomyelitis using a 16S rRNA sequencing approach and to compare the results with conventional culture techniques. In this prospective observational study, we obtained 34 bone samples from patients admitted to our hospital with a moderate–severe diabetic foot infection. We analysed the distribution of the 16S rRNA gene sequences in the bone samples, using an Illumina MiSeq Personal Sequencer. We compared the genera that were detected with the cultured pathogens in the bone samples with conventional techniques. In the 23 samples that had positive results with both techniques, *Staphylococcus*, *Corynebacterium*, *Streptococcus* and *Propionibacterium* spp. were detected in 20, 18, 13 and 11 samples, respectively. Significantly more anaerobes were detected with 16S rRNA sequencing compared to conventional techniques (86.9 % vs. 23.1 %, *p* = 0.001) and more Gram-positive bacilli were present (78.3 % vs. 3.8 %, *p* < 0.001). *Staphylococcus* spp. were identified in all of the sequenced bone samples that were negative with conventional techniques. Mixed genera were present in 83.3 % (5 of 6) of the negative samples. Anaerobic and fastidious organisms may play a more significant role in osteomyelitis than previously reported. Further studies with larger populations are needed in order to fully understand the clinical importance of the microbial diversity of diabetic foot osteomyelitis.

## Introduction

Diabetic foot osteomyelitis (DFO) develops in approximately 44–68 % of patients with diabetes mellitus admitted to the hospital with a diabetic foot infection (DFI) [1] and is the leading cause of amputation among such patients [2]. The microbiologic spectrum of DFO seems to be similar to deep diabetic foot soft tissue infections [3] and primarily consists of Gram-positive bacteria, especially *Staphylococcus aureus* and beta haemolytic streptococci [4, 5]. Anaerobic pathogens are generally uncommon, with some studies reporting that only 3–14 % of infections involve anaerobes [6]. More recent studies indicate that 46–85 % of DFO are monomicrobial [7, 8]. However, conventional culture techniques focus on organisms easily cultured using traditional microbiological evaluations and are limited by the time required for organisms to grow [9]. The phenomenon that only a small percentage of microorganisms grow on agar plates has been known as the ‘great plate count anomaly’ since the early 20th century [10]. Little is known about the diversity of bacteria in DFO and the contribution of anaerobic and fastidious organisms to these infections [11]. This study aimed to better characterise the bacterial ecology of DFO using a modern 16S ribosomal ribonucleic acid (rRNA) gene sequencing approach.

## Materials and methods

### Patient population

We consecutively obtained 34 bone samples from patients admitted to our hospital with moderate–severe DFI according to the diabetic foot infection classification of the Infectious Diseases Society of America (IDSA) [12]. We included patients who were 21 years or older and had high suspicion of DFO based on their IDSA classification. Exclusion criteria included other infectious diseases, active, previously diagnosed DFO in the study foot, immunosuppressive therapy, organ and/or haematological malignancies, and end-stage renal disease requiring dialysis. We performed a percutaneous biopsy using a 16 gauge Jamshidi needle introduced at least 2 cm from the ulcer site [6] (*n* = 7) or we obtained intraoperative bone samples from the patients that required surgical debridement or amputation (*n* = 27). We sent the obtained bone samples to the laboratory for conventional culturing and histopathological tests. We used our hospital microbiology laboratory’s established protocol for anaerobic sampling and transport. The bone specimens were placed in sterile cups without any transport medium and processed within 1 h of collection. Laboratory technicians were kept unaware of the clinical data. We stored a part of the obtained samples promptly at −80 °C until the end of the study. Informed consent was obtained from all individual participants included in the study.

### 16S rRNA gene sequencing

After thawing, we recovered portions of the bone samples using sterile forceps. We extracted genomic DNA using the Roche High Pure PCR Template Preparation Kit (Roche Life Sciences, Indianapolis, IN, USA) with a modified lysis step. We lysed our samples by combining 25 mg of sample, 200 μL each of lysis buffer and binding buffer (kit buffers 1 and 2), 500 μL of zirconium oxide beads and a single 5-mm steel bead in a 200-μL screw-cap tube. We shook the tubes using a TissueLyser II (Qiagen, Inc., Valencia, CA, USA) for 5 min at 30 Hz. We continued the extraction using the manufacturer’s instructions. We used the Illumina MiSeq Personal Sequencer (Illumina, Inc., San Diego, CA, USA) in collaboration with PathoGenius Laboratories (PathoGenius, Lubbock, TX, USA) to assess the distribution of 16S rRNA gene sequences. We amplified the samples for sequencing using a forward and a reverse fusion primer. The forward primer was constructed with the (5′-3′) Illumina i5 adapter, an 8–10-bp barcode, a primer pad and the 28F primer. The reverse fusion primer was constructed with the (5′-3′) Illumina i7 adapter, an 8–10-bp barcode, a primer pad and the 519R primer. We used the HotStarTaq Plus Master Mix Kit (Qiagen, Inc., Valencia, CA, USA) for polymerase chain reaction (PCR) under the following conditions: 94 °C for 3 min; followed by 30 cycles of 94 °C for 30 s, 60 °C for 40 s and 72 °C for 1 min; and a final elongation step at 72 °C for 5 min.

In preparation for 16S sequencing, we denoised the DNA to remove short sequences, singleton sequences and noisy reads [13]. With the bad reads removed, we performed chimera detection using the de novo method built into UCHIME [14] to aid in the removal of chimeric sequences [15]. We corrected the remaining sequences base by base to help remove noise from within each sequence. We quality checked and demultiplexed the denoised and chimera-checked reads generated during sequencing. We then clustered these sequences into operational taxonomic units (OTUs) using the UPARSE algorithm [16]. We ran the centroid sequence from each cluster against a database of high-quality sequences derived from the National Center for Biotechnology Information (NCBI) database, March 2015. We used an internally developed Python program that assigns taxonomic information to each sequence to analyse the output and write the final analysis files.

### Statistical analysis

Analysis was performed using the SAS 9.4 statistical package. The data were presented as number of patients (%). Differences between both culture techniques were measured using the McNemar’s test. *p*-Values <0.05 were considered statistically significant.

## Results

Of the 26 bone samples that grew pathogens with conventional culturing techniques, three did not sequence. The three samples that did not sequence grew *Stenotrophomonas maltophilia* (*n* = 1), *S. aureus* (*n* = 1) and *Enterobacter cloacae* (*n* = 1) with conventional culturing. All three samples were monomicrobial infections. Table 1 presents an overview of all the genera that were sequenced and occurred in at least 21.7 % (5 of 23) of the positive bone samples. The table includes the average contribution of each genus to the total bacterial population in those samples as represented as a percentage. *Staphylococcus* spp. were the predominant genus identified in the positive bone samples. Sequences of *Staphylococcus* spp. were detected in 20 of the 23 samples, with an average contribution of 28.6 % to the total bacterial population. The most prevalent populations of Gram-positive cocci identified after that were, in order, *Corynebacterium* (*n* = 18), *Streptococcus* (*n* = 13) and *Propionibacterium* spp. (*n* = 11). Facultative anaerobes included *Actinomyces* and *Helcococcus* spp. in 6 and 5 of the samples, respectively. Obligate anaerobes such as *Peptoniphilus*, *Finegoldia*, *Anaerococcus*, *Clostridium*, *Porphyromonas* and *Prevotella* were detected in 17, 15, 12, 9, 7 and 5 of the samples, respectively. Two of the positive samples were low coverage samples (sequence counts of 9 and 534, respectively). Both of these low coverage samples only had sequences that matched with *Staphylococcus* spp. and grew coagulase-negative staphylococci with conventional techniques.

**Table 1:** Bacterial genera identified with 16S ribosomal ribonucleic acid (rRNA) sequencing in 23 positive bone samples

Genera*	Samples	Avg %	SD	Min–max %
No hit	22	15.6	26.1	0.03–87.1
*Staphylococcus* spp.	20	28.6	34.6	0.17–98.8
*Corynebacterium* spp.	18	7.0	10.7	0.01–33.8
*Peptoniphilus* spp.	17	2.3	3.1	0.01–11.7
Unknown *Firmicutes*	16	13.1	19.5	0.02–55.6
*Finegoldia* spp.	15	8.1	11.8	0.17–44.6
Unknown Clostridiales	14	3.3	8.4	0.02–32.1
*Streptococcus* spp.	13	20.1	19.5	0.03–57.9
*Anaerococcus* spp.	12	8.2	8.7	0.06–27.6
*Propionibacterium* spp.	11	0.9	1.7	0.002–5.0
*Clostridium* spp.	9	0.9	1.1	0.008–3.3
Unknown Dermabacteriae	8	0.1	0.1	0.03–0.3
Unclassified Clostridiales	8	1.1	1.6	0.008–3.9
Unknown Clostridia	8	2.0	2.1	0.03–6.8
*Porphyromonas* spp.	7	1.8	1.7	0.03–4.8
Unclassified Clostridia	7	1.3	1.5	0.004–3.6
Unknown bacteria	7	2.2	5.1	0.01–13.8
*Actinomyces* spp.	6	1.0	1.8	0.003–4.7
*Enterobacter* spp.	6	6.0	11.3	0.10–28.8
*Prevotella* spp.	5	3.2	5.5	0.04–13.0
*Helcococcus* spp.	5	1.2	1.5	0.05–3.8
*Pseudomonas* spp.	5	20.8	42.8	3.90–52.6

*Genera sequenced that occurred in at least 21.7 % (5 of 23) of the positive bone samples. The genera are sorted by the number of bone samples in which they were detected

*Avg %* average percentage each genus contributed to its positive samples; *SD* standard deviation of the percentages; *Min–max %* range of the percentages; *No hit* sequence has no match with the sequences in the NCBI database

Table 2 presents a comparison of the results of both culturing techniques. Only the genera that occurred in at least 21.7 % (5 of 23) of the positive bone samples with the sequencing method are reported. With 16S rRNA sequencing, we found significantly more anaerobic pathogens (86.9 % vs. 23.1 %, *p* = 0.001), significantly more Gram-positive bacilli (78.3 % vs. 3.8 %, *p* < 0.001) and more polymicrobial infections (91.3 % vs. 64.0 %, *p* = 0.125). Also, greater bacterial diversity was seen both in the Gram-positive cocci and the anaerobes.

**Table 2:** Bacterial genera in diabetic foot osteomyelitis (DFO) with the two culturing techniques

Conventional culture techniques		16S rRNA sequencing*	
Pathogens	Overall (%), total number of patients = 26	Pathogens	Overall (%) total number of patients, = 23
**Gram-positive cocci**	20 (76.9)	**Gram-positive cocci**	23 (100.0)
*S. aureus*, total	13 (50.0)	*Staphylococcus* spp.	20 (86.9)
*S. aureus* resistant to methicillin	3 (11.5)	*S. aureus* resistant to methicillin	Not tested
Coagulase-negative staphylococci	11 (42.3)	Coagulase-negative staphylococci	Not tested
*Streptococcus* spp.	6 (23.1)	*Streptococcus* spp.	13 (56.5)
*Enterococcus* spp.	2 (7.7)	*Enterococcus* spp.	0
		Unknown Dermabacteriae	8 (34.8)
**Gram-positive bacilli**	1 (3.8)	**Gram-positive bacilli**	18 (78.3)
*Corynebacterium* spp.	1 (3.8)	*Corynebacterium* spp.	18 (78.3)
**Gram-negative bacilli**	13 (50.0)	**Gram-negative bacilli**	10 (43.5)
*P. aeruginosa*	4 (15.4)	*Pseudomonas* spp.	5 (21.7)
*S. maltophilia*	1 (3.8)	*S. maltophilia*	0
*Proteus* spp.	1 (3.8)	*Proteus* spp.	0
		*Enterobacter* spp.	6 (26.1)
**Anaerobes**	6 (23.1)	**Anaerobes**	20 (86.9)
Facultative anaerobes	3 (11.5)	Facultative anaerobes	17 (73.9)
		*Propionibacterium* spp.	11 (47.8)
		*Actinomyces* spp.	6 (26.1)
		*Helcococcus* spp.	5 (21.7)
Obligate anaerobes	3 (11.5)	Obligate anaerobes	20 (86.9)
		*Peptoniphilus* spp.	17 (73.9)
		*Finegoldia* spp.	15 (65.2)
		*Anaerococcus* spp.	12 (52.2)
		*Porphyromonas* spp.	7 (30.4)
		*Prevotella* spp.	5 (21.7)
		Unknown *Firmicutes*	16 (69.6)
		Unknown/unclassified Clostridia	15 (65.2)
		Unknown/unclassified Clostridiales	22 (95.7)
		*Clostridium* spp.	9 (39.1)
**Polymicrobial infections**	16 (64.0)	**Polymicrobial infections**	21 (91.3)
**Unknown bacteria**	NA	**Unknown bacteria**	7 (30.4)

*Genera sequenced that occurred in at least 21.7 % (5 of 23) of the positive bone samples. Data are number of patients (%)

No pathogens were identified in 8 out of the 34 bone samples (23.5 %) with conventional culture techniques. Two out of those eight negative samples did not sequence either. The genera that were sequenced in the remaining six and occurred in at least 33.3 % (2 of 6) of the samples are presented in Table 3. *Staphylococcus* spp. were detected in all of the negative samples, with an average contribution of 21.8 % to the total bacterial population. One of the negative samples was a very low coverage sample (sequence count 3); all three sequences matched with the *Staphylococcus* spp. sequence derived from the NCBI database. This was the only negative bone sample that had a single genus present.

**Table 3:** Bacterial genera identified with 16S rRNA sequencing in six negative bone samples

Genera*	Samples	Avg %	SD	Min–max %
*Staphylococcus* spp.	6	21.8	39.3	0.05–100.0
No hit	4	49.9	40.8	5.07–97.9
*Corynebacterium* spp.	3	3.8	1.6	1.99–5.0
*Propionibacterium* spp.	3	5.9	9.5	0.35–16.8
*Streptococcus* spp.	3	12.2	15.3	1.37–29.7
*Anaerococcus* spp	3	4.4	5.6	1.12–10.9
*Finegoldia* spp.	3	6.8	3.2	3.14–8.9
*Peptoniphilus* spp.	3	1.9	1.4	1.02–3.5
Unknown *Firmicutes*	3	7.2	9.1	0.03–17.4
*Enterobacter* spp.	3	0.2	0.2	0.02–0.5
Unknown Microbacteriaceae	2	7.6	10.4	0.27–15.0
Unknown *Enterobacter*	2	0.4	0.5	0.02–0.7
*Pseudomonas* spp.	2	18.4	26.0	0.04–36.8
Unknown bacteria	2	0.4	0.3	0.20–0.7

*Genera sequenced that occurred in at least 33.3 % (2 of 6) of the negative bone samples. The genera are sorted by the number of bone samples in which they were detected

*Avg %* average percentage each genus contributed to its positive samples; *SD* standard deviation of the percentages; *Min–max %* range of the percentages; *No hit* sequence has no match with the sequences in the NCBI database

## Conclusions

The primary genus detected in the bone samples of the current study was the *Staphylococcus* spp., both with conventional culturing techniques and with 16S ribosomal ribonucleic acid (rRNA) gene sequencing. Not only was it detected in 89.6 % (26 of 29) of the sequenced samples, its average contribution to the total bacterial population was the highest of all the genera. This is not a surprising result, since nearly every study reported in the North American and European literature identifies *Staphylococcus aureus* as the most common pathogen cultured in diabetic foot osteomyelitis (DFO), followed by *S. epidermidis* [1].

The *Corynebacterium* spp. was the most prevalent population after *Staphylococcus* spp. However, the average contribution of this genus to the total bacterial population appears to be much lower. This fastidious organism has been associated with DFO in previous studies that used traditional culturing methods [17, 18]. In a recent study by Dowd et al. [11], the *Corynebacterium* spp. was even identified as the predominant genus in the individual ecologies of 40 diabetic foot ulcers using a similar sequencing approach. The *Staphylococcus* spp. was only detected in 13 of the 40 debridement samples. However, the pathogenic role of *Corynebacterium* spp. in infections is not well understood and the genus is usually considered a contaminant.

Because DFO is not typically exposed to air, especially if peripheral arterial disease is present, anaerobes may play a bigger role than expected. As has been previously reported in studies using pyrosequencing to characterise bacterial diversity in chronic osteomyelitis of the jaw [19], osteomyelitis was not caused by a single pathogen but by diverse bacteria comprising both aerobic and anaerobic species, including unculturable bacteria with conventional culturing methods. Only 3 out of our 29 sequenced bone samples had a single genus of bacteria present, and all three of these samples had low sequence counts, so they may have been contaminants. Our results show that, by using a 16S rRNA sequencing technique, anaerobes were detected in 86.9 % of the positive bone samples (vs. 23.1 % with conventional techniques). The number of anaerobes seems to be largely dependent on the culturing method. In studies by Senneville et al. [6] and Ertugrul et al. [20], obligate anaerobes were identified in only 3 % and 5 % of patients, respectively, after optimising the culturing methods.

All of the patients enrolled in the study were admitted to our hospital with moderate/severe Infectious Diseases Society of America (IDSA) infections that required antibiotics and surgery urgently per IDSA treatment guidelines. Therefore, a limitation of our study design is the high pre-test probability of osteomyelitis and the relatively small number of negative subjects. In addition, we did not have a ‘wash-out’ period with no antibiotic therapy before bone cultures were obtained. While this convention is discussed in the medical literature, there is no direct evidence that it affects the results of either culture technique. However, previous antibiotic therapy may have favoured the results of 16S rRNA sequencing, since pathogens did not need to be viable in order to be detected.

The use of advanced biological molecular technology is of particular interest in DFO, wherein the chronicity of the infection and the adhesion of bacteria in a sessile phenotype may make it difficult to culture these pathogens [21]. The diversity of the bacterial population may contribute to the poor success rates of medical treatment of DFO [22–24]. Studies report prolonged treatment courses with antibiotics in non-surgical cases ranging from 42 to 90 days [25], up to even 40 weeks [26], as well as considerable variation in success (57–70 %) [25]. Culture-specific antibiotic treatment has been reported to provide a higher rate of treatment success compared to empiric therapy [7]. A better understanding of the bacterial diversity in DFO will provide new insights to redirect therapy and might improve clinical outcomes in the future [27].

## Abbreviations

DFI: Diabetic foot infection
DFO: Diabetic foot osteomyelitis
IDSA: Infectious Diseases Society of America
NCBI: National Center for Biotechnology Information
OTU: Operational taxonomic units
rRNA: Ribosomal ribonucleic acid
